# West Texas Medical Association

**Published:** 1902-02

**Authors:** W. B. Russ

**Affiliations:** Secretary


### West Texas Medical Association.

Regular meeting, San Antonio, January 2.

A committee was appointed, with Dr. Paschal as chairman, and instructed to make up a list of practitioners suspected of not having complied with the law and to submit same to the county attorney together with a demand in writing that he give the matter his immediate attention.

The secretary reported that two hundred blank membership certificates, engraved on the best parchment paper obtainable and as large as the ordinary college diploma, had been ordered and would be ready for distribution in a short time.

A motion to invite the Texas State Medical Association to meet in San Antonio was carried by a unanimous vote. [See January number.]

Dr. Kingsley read a well prepared paper on "Irregular Types of Appendicitis." Dr. Burg lead the discussion.

Captain Charles F. Mason, M. D., reported two interesting cases of appendicitis to illustrate that symptoms do not always indicate the gravity of the disease. Drs. Young and Goeth also discussed Dr. Kingsley's paper.

Dr. Paschal presented a number of pathological specimens of unusual interest.

W. B. Russ, M. D., Secretary.

